# Progress of Discovery in the Nervous System and in Medical Ethics

**Published:** 1856-05

**Authors:** Bennet Dowler

**Affiliations:** New Orleans


### SELECTIONS.

### *Progress of Discovery in the Nervous System and in Medical Ethics.*

(From the New Orleans Medical and Surgical Journal, March, 1856.)

"FOREIGN.—*Functions of the Spinal Cord.*—Experiments made by Dr. Brown-Séquard during the past summer, and presented to the Biological Society of Paris,—a report upon which has been made by a commission appointed for the purpose—threaten to overthrow the present received notions of the functions of the spinal cord, as they are taught in our books. One experiment made in July last, according to M. Broca, the reporter of the commission establishes beyond doubt the following points: 1. That the laying bare of the dura mater, and of the spinal cord itself, leaves the sensibility and motion of the posterior column unaffected. 2. That the sensibility persists even after the section of the posterior columns, called the sensitive columns, of the spinal marrow, and that, consequently, these columns are not indispensable to the transmission of sensitive impressions. 3. That, far from abolishing the sensibility, the section of these pretended sensitive columns is accompanied by hyperæsthesia of the lower extremities. 4. That after such a section, the caudal extremity of the spinal cord is more sensitive than the cephalic, which reverses all of our present knowledge of the direction of nervous currents. 5. Finally, the gray portion of the cord is itself insensible."—*L'Ab. Méd.*, Sept. 5, 1855.—*New-York Med. Times*, Feb. 1856.

The Boston Medical and Surgical Journal for Feb. 1856, hails with pleasure M. Brown-Séquard's researches, thus: "M. Brown-Séquard's discoveries of the functions of the spinal marrow: seldom has the scientific world been taken more by surprise than when M. Brown-Séquard announced his recent discoveries relative to the functions of the spinal marrow. Whatever may be wanting to complete our knowledge of the action of this portion of nervous system, the brilliant investigations of Sir C. Bell, seemed to have set at rest forever the question as to the particular fibres which communicate motion to the muscles, and sensation to the brain * * * M. Brown-Séquard ascertained that the nervous disturbance following the opening of the spinal canal was caused by the loss of blood and by the pain and shock consequent upon the operation. By operating in such a manner as to prevent a great flow of blood, and by allowing the animal time to recover from the depressing effects of the operation, he found that both sensation and motion returned to the posterior extremities in almost, if not quite, their original degree.

"Thus enabled to experiment upon the cord in a normal state (as far as its functions were concerned,) he proceeded to isolate various portions of the different columns by sections made with extreme care, and demonstrated a series of laws relative to the spinal functions, the principal of which are the following:

1. The posterior columns may be divided without destruction either of sensation or motion.

2. Sensation and motion are destroyed when the grey substance is cut across.

3. Integrity of the antero-lateral columns does not interfere with the loss of motion, nor does integrity of the posterior columns prevent loss of sensation.

4. Division of the posterior fibres of the cord, so far from abolishing sensation in the parts to which these fibres are distributed, appears, on the contrary, greatly to increase it.

5. When the posterior columns are divided, sensation continues to be transmitted between the lower portion and the grey substance, which transmits the impression to the sensorium by means of fibres decending from the upper portion, and joining obliquely the grey substance below the point where the section is made.

"Our limits forbid us to detail the experiments upon which the above conclusions are founded. They have been repeated over and over again with the same results, in the presence of a committee appointed by the *Société de Biologie*, consisting of M. M. Claude Bernard, Bouley, Broca, Giraldés, Goubaux and Velpein, to whom was referred M. Brown-Séqard's memoir, and who were entirely satisfied with his conclusions. The interesting report which they made to the Society is the most convincing evidence of M. Brown-Séquard's skill as an experimenter and his eminence as a physiologist."

At present, neither time, nor the limits of this Journal will permit the editor to translate and copy from the French Journals the reported researches above alluded to, a few general remarks in relation to the same, may not, it is hoped, prove unacceptable to the right-thinking reader, seeing that at last "the world is taken by suprise."

Sir C. Bell repudiated experimental vivisections and electrical excitations declaring he had performed but few experiments, and did not rely on them to prove his (pretended) discovery in the nervous system. The foundation of his theory rests upon the assumption that, it is impossible for one set of nerves to perform two functions, that is to say, sensation and voluntary motion, as if the same nerve might not, for all that is known to the contrary, perform two, three, or many functions as readily as one. Yet it is neither philosophically or physiologically correct to assume for any single isolated tissue of the human body all independent and exclusive power of acting apart from its associated tissues and fluids, unless, indeed, it being the muscular tissue which, contrary to the doctrine of Bell, possesses the inherent power of contractility exclusive of the nerves, &c. This theory assumes as a fundamental maxim, that sensation and motion are due to the nerves, to separate and distinct nerves; that is to say, the sensiferous and the motiferous. Hence, the functions being assumed, the anatomy is also assumed, which serves to pave the way for other assumptions still more extraordinary. Thus the sensational nerves have no sensation, being mere conductors. Nothing more! The motiferous nerves possess no force, no motion, being mere conductors. Nothing more! It is further assumed that there is a spot, never seen, either by the eye, or the microscope, somewhere in the brain, which is called the *sensorium*—*a terra incognita*, not *a terra innominata*, and being wholly unknown, theorists may predicate a thousand things of it which cannot be disproved, such as that, this sensorium which is neither known by intuition, nor by anatomy, receives by one set of nerves certain impressions, and transmits by another set, volitions, &c. Thus the word sensorium is an admirable thing where all knowledge fails.

This system of physiology is not only unsupported by anatomy, (yet, it was anatomy alone Bell relied on), but it is directly contradicted in conduct, if not in books, by the intuition of every sane man. A whitlow in the hand, a corn on the toe, or a boil on the surface, does not produce a pain in the unknown spot in the brain, but in the known spot on the surface. Who would not swear to this in open court?

To cap the climax of all these assumptions, the brain or sensorium is said to be wholly insensible, which, indeed, is not very far from the truth: for, in vivisecting an animal, it shows comparatively little sign of pain until the knife approaches the origin of the nerves arising from the brain. This insensibility of the brain or sensorium, exceeding by far that in the spinal cord, is very unfortunate for the central sensationalists, because the sensiferous nerves is, according to them, but an insensible conductor to an insensible sensorium, sensation being thereby rendered impossible, unless *the-is-not*, is more potent than the *is*. They dethrone entities in order to inaugurate nonentities.

The assumption of Bell,* Magendie, and all the world, that, because the nerves have double roots they must consequently have double functions entirely distinct, is just as convincing an argument for double functions in a tooth having two roots, or in the biceps muscle having two heads, not to name many organs more distinctly duplex, as double eyes, ears, lungs, kidneys, testes, ovaries, &c.

The two separate and wholly distinct sets of nerves said to be distributed to the organism, cannot be discovered even by the microscope, as the fanatical propagandists of this school themselves admit. Yet they found their books of physiology, pathology, and therapeutics upon this system, and fight for it with the zeal of martyrs. Is this wise? moral?

It is now more than a dozen of years since the writer of these lines began to publish his experimental researches and inquiries concerning this system. Hundreds of human beings were submitted to experiment immediately after death; many vivisections of animals were performed before numerous medical witnesses competent to judge of their validity and fairness—witnesses who agreed in their testimony, and had no rational motives to deceive. Animals were decapitated, their spinal cords divided transversely and longitudinally, without destroying sensation and voluntary motion; the posterior and anterior roots of the spinal cord were irritated, divided, and destroyed; the nerves were followed to their distributions, and dissected away, as the readers of this journal and other journals know, showing that all of the fundamental principles of Bell and Magendie, concerning the *two* sets, as well as Hall's *four* sets of nerves, were erroneous—that the muscles possess an inherent power of contraction; that both the posterior and interior nerve-roots give indifferently the signs of motion and sensation; that the twitching motions described by Bell and Magendie as exclusively pertaining to the anterior

*Both Bell and Magendie were later by several years, than Walker, in the assumption of double nerves and functions.

root, can always, contrary to their theory, be more fully produced after separation from the cord than before, by following the nerve towards its distribution; that the spinal cord can be divided in three or four places, and that the head may be removed without destroying sensation and voluntary motion, and that sometimes, in the alligator at least, the head on the one hand, and the separated body on the other, act in a voluntary manner each for itself, giving all the rational indications that could be expected of an animal, as purpose, feeling, intelligent motion, &c.

It is however in the decapitated body that all the conceivable phenomena characterising sensation and voluntary motions are the most strikingly manifested, often for hours, with the exception of such phenomena as appertain exclusively to the special senses, as hearing, seeing, &c., the body acting of course like a blind animal until informed by touch.

Pamphlets, though few in number, describing these experiments, and announcing the deductions drawn from them were sent without delay to the principal capitals of christendom. And now, anno 1856, the latest mails bring the *unexpected news*, chiefly from *Paris*, showing that within the *last six months* experiments have been made, and pathological cases have been observed, which, though less striking, and less varied than the old New Orleans experiments, are received with loud acclaim in America, as overthrowing Bell's theory, and forming a new æra in physiology. Royalty inaugurated Bell with knighthood and a pension, and now the Imperial Academy of Medicine inaugurates M. Browne-Séquard as a discoverer, without recognizing in the remotest degree, the *priority* of New Orleans. (See *L' Union Méd.* Oct. 6, '55; Sept. 8th, '55; *Gaz. Hebdom. de Méd.* Sept. 14th; Oct. 5th; Nov. 2d, 1855; also, the case, and post-mortem examination reported in *L' Union Méd.*, by M. Laboulbène, which occupied nearly the entire number of the Journal for Dec. 15th, 1855. "'Tis distance lends enchantment," &c.

M. Brown-Séquard is a distinguished French physiologist, who of late years lectured in Boston, New York, and in other parts, and who, for a few weeks during last year, occupied the chair of physiology in the Medical College of Richmond, Virginia. His compatriots of the Imperial Academy of Medicine reported without delay*—without waiting or wishing to hear from Trans-Atlantic realms; they gave him their cordial approval, as independent philosophers always should do, however humble may be the laborer in the field of science. The Academy had, as yet, scarcely ceased to insist on Magendie's claims to priority of discovery over Bell—the members of that learned body, perhaps, had not pronounced the funeral oration upon the former, their illustrious defunct associate, when they pronounced upon M. Brown-Séquard's important researches, as overthrowing Bell and Magendie's theory—researches which, though but confirmations, not to say plagiarisms of another (so far as the nervous system is concerned,) will inaugurate fundamental principles, of high importance in physiology, not to say psychology.

BENNET DOWLER, M. D.

*New Orleans*, *March* 1, 1856.

---

* In Paris the rule seems to be that whatever claims to be an original contribution to, or discovery in, science, is examined into, reported upon, and adopted if found true at the weekly sitting of the Academy, without delay. Has the ethical law which, as it were, obliges one to accept the truth in Imperial France, a velocity and force 624 times greater than in Republican America? or is Ethical arithmetic in different places as 1 week to 12 years=624 weeks?

